# Development and Validation of a Specific Self-Efficacy Scale in Adherence to a Gluten-Free Diet

**DOI:** 10.3389/fpsyg.2018.00342

**Published:** 2018-03-16

**Authors:** Ricardo Fueyo-Díaz, Rosa Magallón-Botaya, Santiago Gascón-Santos, Ángela Asensio-Martínez, Guillermo Palacios-Navarro, Juan J. Sebastián-Domingo

**Affiliations:** ^1^Aragon Institute of Health Sciences IACS, Zaragoza, Spain; ^2^Department of Psychology and Sociology, University of Zaragoza, Zaragoza, Spain; ^3^Department of Medicine, Psychiatry and Dermatology, University of Zaragoza, Zaragoza, Spain; ^4^Department of Electronic Engineering and Communications, University of Zaragoza, Zaragoza, Spain; ^5^Department of Digestive Diseases, Hospital Royo Villanova, Zaragoza, Spain

**Keywords:** celiac disease, self-efficacy, adherence to a gluten-free diet, perceived quality of life, empowerment

## Abstract

The aim of this study was to develop a scale to assess the levels of specific self-efficacy in order to enhance adherence to a gluten-free diet and the life quality of celiac patients. Celiac disease is a chronic small intestinal immune-mediated enteropathy precipitated by exposure to dietary gluten in genetically predisposed people. The only treatment is a strict lifelong gluten-free diet. Within the framework of Social Cognitive Theory, expectation of self-efficacy is understood as the degree in which a person believes himself to be capable of performing a certain task (e.g., adhering to a gluten-free diet), a construct which has been widely studied in its relation with adopting healthy behaviors, but scarcely in relation to celiac disease. A validation study was carried out in various stages: preparation of the protocol; construction of the questionnaire and a pilot run with 20 patients; validation of the scale with 563 patients and statistical analysis. A 25-item scale was developed. Feasibility was excellent (99.82% of participants completed all the questions). Factorial analysis pointed to the existence of five factors that explained 70.98% of the variance with a Cronbach alpha of 0.81 for the scale overall and between 0.64 and 0.90 for each factor. The scale showed a Spearman's Rho coefficient of 0.279 with the General self-efficacy Scale. This easily administered scale provides good psychometric properties for evaluating specific self-efficacy of celiac patients in adhering to treatment. It seeks to be the first scale that provides not only a measurement of specific self-efficacy in celiac disease, but also to determine its levels for each of the areas as a first step toward designing interventions of self-management and empowerment programs to cope with the disease.

## Introduction

Celiac disease (CD) is a chronic small intestinal immune-mediated enteropathy precipitated by exposure to dietary gluten in genetically predisposed people (Ludvigsson et al., 2013). Numerous studies report a prevalence of between 1:67 and 1:250, for the USA and Europe (Leffler et al., 2008), while 1:100 is a widely accepted figure (Catassi et al., 2007). The only valid treatment known today is a lifelong strict gluten-free diet (GFD). Despite the benefits of a GFD, rates for strict adherence range from 42 to 91% depending on definition and method of assessment (Hall et al., 2009).

Within Social Cognitive Theory, Bandura defines self-efficacy as “beliefs in one's capabilities to organize and execute the course of action required to produce given attainments” (Bandura, 1997, p.3). Thus, the expectation of self-efficacy has been widely studied in many spheres such as physical activity (Schwarzer et al., 2008), tobacco addiction (Hendricks et al., 2010), multiple sclerosis (Chiu et al., 2011), or patients with arthritis (Lorig et al., 2014). However, it has received scant attention for celiac disease (Ford et al., 2012). High levels of self-efficacy are related to the perception of well-being and adherence to healthy eating (Luszczynska et al., 2005). Recently, self-efficacy has been linked to better adherence to GFD and better quality of life in celiac patients (Ford et al., 2012).

Qualitative studies show that celiac patients have to cope with problems mainly in five areas: eating in the workplace, shopping, traveling, eating out, and eating at home with others (Sverker et al., 2005). These difficulties can lead to negative emotions and affect relationships. Celiac patients with low self-efficacy can find eating and drinking situations potentially stressful and, hence, are not able to keep to their GFD in such circumstances, or if they are able to, it is at the cost of a lower life quality (Leffler et al., 2008).

There are a few questionnaires aimed to evaluate self-efficacy in a general way: Sherer's General Self Efficacy Scale (Sherer et al., 1982), recently translated into Spanish and validated (Herrero et al., 2014), Schwarzer's General Self Efficacy Scale (Schwarzer and Jerusalem, 1995), and the New General Self Efficacy Scale (Chen et al., 2001). All three scales provide solid psychometric information (Scherbaum et al., 2006) but none of them provide a specific measure of self-efficacy. Bandura (1997) emphasizes the convenience of specifically evaluating self-efficacy expectation and in a way that is closely linked to the demands of a particular situation, rather than making general evaluations of that situation. We have not found any scale to assess specific self-efficacy in celiac patients.

More recently, Schwarzer has proposed the Health Action Process Approach (HAPA model) as an explanatory framework for adherence to healthy habits (Schwarzer et al., 2011) in which self-efficacy plays a key role.

Traditional explanatory models of change fail to explain the gap between intention and action. The HAPA model distinguishes between pre-intentional motivational process and post-intentional volitive processes that lead to healthy habits. We think that this model is useful to explain adherence to GFD. The HAPA model is described in two-phases: a pre-intentional motivational and a post-intentional volitional phase. It is in the initial motivational phase, when the individual still needs to develop the intention to acquire a healthy habit (e.g., adherence to a GFD). In this phase, risks are seen as threatening but unlikely, especially by asymptomatic patients, and not important enough to build an intention. Conversely, they are important enough to motivate the patient toward a contemplation stage for the evaluation of the capabilities needed to take up a GFD (social skills, facing temptations, etc.) and negative consequences (giving up to certain foods, changing habits or extra work associated with the diet). In the same manner, positive consequences are important at this motivational phase (for example, a healthier diet or symptomatology improvement). According to this model, in this phase, high self-efficacy beliefs, together with positive outcome expectations, play a major role and both are necessary to develop an intention. But the development of an intention is not enough. Once developed, in a second phase, this intention needs to be turned into action and, finally, into a strict adherence for which self-regulation skills and strategies are required. In this volitional phase, planning, and self-efficacy beliefs to face transgression play a central role.

Using this model, we developed a scale to assess specific self-efficacy as a determinant in adherence to GFD and subsequently be able to investigate its impact on the quality of life in celiac patients.

## Methods

A multi-phase prospective, observational study was designed in various stages (Grau, 1995): preparation of the protocol, construction of the questionnaire, and pilot run and validation of the questionnaire.

### Phase 1: preparation of a protocol

Prior to the study, the research team drew up a protocol (Fueyo-Díaz et al., 2015). The study population was celiac patients who had been prescribed a lifelong GFD. A minimum age of 12 years was fixed as this is the age at which primary education ends and adolescents starting secondary education have to start managing their diet on their own. It is also assumed that by this age the patient has acquired sufficient language skills to be able to understand and reply to the questionnaire. A sample size of 10–15 patients per item was estimated as appropriated for the validation phase (Kline, 1998).

### Phase 2: construction of the scale and pilot study

The research team created an initial questionnaire based on the Spanish version of the General Self-Efficacy Scale (GSES) (Baessler and Schwarzer, 1996) and follow the recommendations for constructing this type of scale (Bandura, 2006). This first questionnaire contained 80 questions, distributed in the areas identified by Sverker: (1) Shopping: the celiac patient can often experience problems with labels or when asking for gluten-free products over the counter, etc. (2) Travel: how they cope with the diet when using a foreign language or in a place where customs are different, etc. (3) Eating at home with others: this section aims to evaluate those situations in which the behavior of others has to be corrected when this may suppose a risk, identifying oneself as a celiac sufferer and not seeming brusque or rude when refusing food offered by others. (4) Eating out: this section examines aspects such as rejecting dishes that might not be safe. (5) Eating in the workplace or at school: here, social situations at work are explored, such as finding gluten-free options at business or school celebrations.

The initial questionnaire was then analyzed by a team of experts in celiac disease comprising researchers, physicians, psychologist, dietitians and patients. The number of items was whittled down to the 40 most significant. In order to analyze the face and construct validity, the new version of the questionnaire was then studied and assessed by a second team of experts. The opinions and comments of the experts helped to add, remove, or clarify the items and to decide on their inclusion in the definitive scale. After this second team of experts the number of items remained unaltered with 40 items. The scale was constructed to allow responses for all items from 0 (not at all able) to 10 (totally able) in order to evaluate the degree of self-efficacy respondents experienced in each of the situations proposed. To get both the scale and subscale scores we took the mean value of the answers. Although 99.82% of participants completed all the questions, missing data were completed with the mean value obtained by the rest of the subjects in the missed particular item. In clinical settings, although we recommend to use only completely filled questionnaires, missing values can be substituted by the mean in the subscale. A pilot study of the new version was carried out with 20 patients to check the relevance and comprehension of the questions selected.

### Phase 3: questionnaire validation

The study was advertised through patients' associations and invitations to participate were sent out to their associates.

The questionnaire was administered on line alongside the Spanish adaptation of the GSES (Baessler and Schwarzer, 1996). Along with these items, we incorporated sociodemographic variables on place of residence, the year of diagnosis, time on GFD, and age.

The questionnaires were completed anonymously and then returned to the research team. The questionnaire returned two types of measurements: an overall score for the questionnaire and scores for each of the five areas, so enabling the evaluation of specific self-efficacy in each of the areas analyzed.

The questionnaires were collected between June and September 2014.

### Phase 4: statistical analysis

Construct validity was determined by factorial analysis (principal components with a VARIMAX rotation) based on eigenvalues >1 criterion (Kleinbaum et al., 1978). We included the necessary factors to obtain a capacity to explain approximately a 70% of the variance. The relevance of the factorial analysis was evaluated using a correlation matrix among the variables, the Barlett sphericity test to study the identity of the correlation matrix and the Kayes, Meyer, and Olkin statistic to measure sample suitability.

Concurrent validity was calculated with Spearman's Rho coefficient with the GSES scale for each of the areas and for the questionnaire as a whole while the Cronbach alpha was used to study overall reliability of the questionnaire and for each of the scales. Multiple comparisons were performed by one-way ANOVA and *post-hoc* evaluations by Scheffe' test. Kruskal–Wallis and U Mann–Whitney tests were performed when necessary. The SPSS v.21 program was used for the statistical analysis and 0.05 was taken as being statistically significant throughout.

This study was approved by the Aragon Scientific Research Ethics Committee (CEICA), registered under number PI 14/0011. The research team obtained written and informed consent from the participants, or their legal guardians.

## Results

### Participants

Five hundred and sixty-three valid 40-item questionnaires were collected. Patients were aged from 12 to 72 years *(M:* 37.37; *SD:* 13.80) and 77.8% were females. Age at diagnosis ranged from 1 to 69 (*M:* 28.15; *SD:* 15.26). Patients had been following a GFD from <1 year to 59 *(M:* 8.77; *SD*: 8.74) (Table 1). Feasibility was excellent, 99.82% of participants completed all the questions. There was no floor effect, and ceiling effect was low (3.9%).

**Table 1:** Characteristics of participants.

**Variable**	
Sex (% female)	77.8
Age % (<18/18-35/36-65/>65)	13.5/29.8/55.2/1.4
Years on GFD (<1/1-3/4-5/<4	6.4/23.9/14.6/55
% Member of patients' association	97.8

### Study of the suitability of the factorial analysis

The relevance of the factorial analysis was evaluated using a correlation matrix among the variables. The associated *p*-value for the Barlett Sphericity Test was *p* < 0.001, indicating that there was, indeed, a relation between the items, which was a guarantee of the technical suitability of the factorial analysis. Finally, the KMO value was 0.934, which is well above the recommended 0.75. The obtained results showed that there was enough support to perform the pertinent factorial analysis.

### Determination, extraction, and interpretation of factors

Prior to the final factorial analysis, items that fulfilled the following criteria were eliminated: (1) any item that had >5% missing data; (2) any item that correlated poorly with the total scale (i.e., item-to-total correlation <0.40) and thus measured a different construct; (3) pairs of redundant items (i.e. an item-item correlation >0.75). The final questionnaire comprised 25 of the original 40 questions. These were grouped in five areas (Table 2).

**Table 2:** Rotated components matrix.

**Items**	**Factor 1**	**Factor 2**	**Factor 3**	**Factor 4**	**Factor 5**
**SHOPPING**					
1. When I have to ask people to clean machines, utensils or surfaces, for example at the butcher's.	0.405	0.259	−0.107	**0.370**	0.195
2. When shopping I have to reject a product that may not be safe for me.	0.131	0.218	−0.061	**0.613**	0.164
3. When I have to resist buying something that looks very appetizing but may contain gluten.	0.045	0.163	0.292	**0.685**	0.119
4. My belief in my ability to adhere to the recommendations of doctors and associations when shopping is …	0.180	0.220	0.240	**0.631**	0.047
**TRAVELING**					
5. When I'm traveling and I have to find a gluten-free meal and I have not brought my own food.	0.239	**0.802**	0.029	0.098	0.317
6. When I'm traveling in places I know and I have find a gluten-free meal and I have not brought my own food.	0.262	**0.785**	0.068	0.114	0.246
7. When I have to find a gluten-free meal when traveling in unknown places and I have not brought my own food.	0.245	**0.833**	0.047	0.090	0.237
8. When I travel abroad but speak the language and have not brought my own food.	0.243	**0.838**	0.034	0.075	0.187
9. My confidence in not abandoning my gluten-free diet when visiting a city and I want to go to restaurants sample the typical food there…	0.046	**0.660**	0.278	0.320	−0.055
10. When I'm traveling and I have to find a gluten-free meal and I have not brought my own food.	0.076	**0.653**	0.265	0.322	0.018
**EATING AT HOME WITH OTHERS**					
11. Overcome the temptation to abandon the gluten-free diet when the house is full of appetizing food and drink.	0.086	0.072	**0.655**	0.406	−0.043
12. Rejecting food or a present that may contain gluten because I don't wish to seem rude which other people bring and invite me try.	0.139	0.037	**0.585**	0.537	0.054
13. When cooking a meal for others that may contain gluten and I want to join in.	0.111	0.144	**0.754**	0.066	0.053
14. When someone offers me something to try from their plate and which may contain gluten.	0.133	0.045	**0.820**	0.085	0.223
**EATING OUT**					
15. Informing a server in a restaurant that I am a celiac sufferer when on my own.	**0.877**	0.239	0.097	0.124	0.095
16. Informing a server in a restaurant that I am a celiac sufferer when with friends.	**0.901**	0.208	0.126	0.126	0.135
17. Informing a server in a restaurant that I am a celiac sufferer in the company of others who are not in my confidence.	**0.893**	0.231	0.109	0.084	0.170
18. When I want to relax and enjoy a meal in a quiet restaurant.	**0.758**	0.174	0.315	0.125	0.130
19. When refusing a dish that has been brought to my table in a restaurant because I think it may not be sufficiently safe.	**0.494**	0.162	0.173	0.405	0.218
20. When ordering a meal in a restaurant with sufficient guarantees that it is compatible with a gluten-free diet.	**0.618**	0.134	0.219	0.425	0.285
21. When I am alone, taking out and eating food I had prepared at home in case there was no gluten-free option.	**0.402**	0.055	0.170	0.553	0.308
**WORK OR STUDIES**					
22. When identifying myself as a celiac sufferer in the business or students' meal.	0.673	0.155	0.176	0.209	**0.396**
23. Finding gluten-free food and drink in the work or study place.	0.353	0.242	0.168	0.176	**0.717**
24. Finding a gluten-free meal on a business trip or on an excursion.	0.304	0.337	0.153	0.227	**0.744**
25. Finding gluten-free food and drink at business or student celebrations.	0.184	0.315	0.192	0.225	**0.777**

*Bold values show relevant items associated with each factor that was selected for the subscale*.

Since the factorial analysis was confirmatory, we forced the model to extract five factors, explaining 70.98% of the total variance. The rotated components matrix (Table 2) shows the coefficients associated to each item, and its area or factor after a VARIMAX rotation.

The matrix shows the following structure: factor 1, “eating out,” explains 43.23% of the variance; factor 2, “traveling” explains 9.57% of the total variance; factor 3, “eating at home with others” 8.60% of the variance; factor 4, “shopping,” accounts for 4.97% of the variance. Lastly, factor 5, “work/studies,” explains 4.68% of the variance. The higher the factor loading, the higher the association of the item with the factor grouping. The highest values for each item are shown in boldface with two exceptions: Although item 1 “When I have to ask people to clean machines, utensils or surfaces, for example at the butcher's” and item 22 “When identifying myself as a celiac sufferer in the business or students' meal” load onto factor 1, we have considered their second highest load to keep them in their original areas, due to their meaning and importance in dealing with GFD and we will wait for future studies to either eliminate them or rewrite them and assign them to other area.

### Determination of the psychometric properties

In order to analyze reliability, Cronbach's alpha was calculated for all the areas. The scores ranged from 0.64 for “shopping” to 0.90 for “traveling,” indicating a good reliability of the structure obtained from the factorial analysis, as can be seen in Table 3. The alpha coefficient for the whole scale was 0.81.

**Table 3:** Cronbach alpha.

**Area**	**Cronbach alpha**
Shopping	0.644
Traveling	0.904
Eating at home with others	0.842
Eating out	0.858
Work or studies	0.881

The criterion validity was analyzed using the non-parametric Spearman's Rho correlation coefficient with the General Self-Efficacy Scale (GSES), which was administered alongside the questionnaire. The value was 0.279, indicating a low but significant correlation (Table 4).

**Table 4:** Spearman's Rho correlation coefficients.

**Area**	**Spearman's correlation coefficient**
Shopping	0.233^*^
Traveling	0.217^*^
Eating at home with others	0.166^*^
Eating out	0.241^*^
Work and studies	0.220^*^
Total	0.279^*^

* *Significant in all the cases for p < 0.01*.

### Specific self-efficacy

Table 5 shows the results for specific self-efficacy for each of the areas. The areas in which most patients have difficulties adhering to a GFD is traveling, followed by shopping, and work. Scores below 7 are considered to represent low self-efficacy and scores below 5 indicate very low self-efficacy expectations. Scores below 70% of the highest score can be considered low self-efficacy. This is a valid method for determining a cut-off point for high and low self-scores in Likert type survey questionnaires (Child, 2006; Hicks and McFrazier, 2014).

**Table 5:** Results Celiac-SE.

		**Shopping**	**Travel**	**Eating at home with others**	**Eating out with others**	**Work and studies**	**Total**
Mean		8.52	7.35	9.30	8.90	8.86	8.59
Standard deviation		1.46	2.20	1.13	1.46	1.60	1.32
Percentiles	25	7.80	6.00	9.13	8.47	8.33	8.00
	50	9.00	7.83	9.75	9.47	9.67	8.95
	75	9.60	9.17	10.00	9.93	10.00	9.55
Cut-off point	<5	3.7%	16.9%	1.4%	2.9%	3.9%	2.8%
	<7	15.2%	38.3%	6.2%	9.6%	13.2%	11.5%

Differences were found for sex (*p* = 0.030) and for years on GFD between those with <1 year of experience and those with more than 5 years (*p* = 0.044). Differences for age were found between the adult group and young adults group (*p* = 0.000) and young adults participants and seniors (*p* = 0.000). No differences were found for age at diagnosis (Table 6).

**Table 6:** Results Celiac-SE by sex, age, and experience.

**SEXO**	***N***	**Mean**	***SD***	***p***
Male	119	8.89	1.05	0.030
Female	438	8.64	1.36	
**AGE GROUP**				
Teens <18	75	8.74	0.96	
Young adults (18-35)	168	8.32	1.42	
Adults (36-65)	311	8.86	1.28	0.000^*^
Seniors >65	8	9.70	0.24	0.000^*^
Total	562	8.70	1.31	
**YEARS ON GLUTEN FREE DIET**				
<1 year	35	8.11	1.86	
1–3 years	131	8.68	1.30	
4–5 years	80	8.67	1.30	
>5 years	300	8.78	1.22	0.044^**^
Total	546	8.70	1.31	

* *Significant differences (p < 0.05) with young adults group*.

** *Significant differences (p < 0.05) with less than 1 year gluten free diet subjects*.

## Discussion

Self-efficacy expectation plays a central role in healthy habits and adherence to treatments (DiClemente et al., 1985, 1995; Brus et al., 1999; West et al., 1999; Lorig and Holman, 2003; Schwarzer et al., 2008), but this has not been studied in relation to celiac disease, in part because of the lack of specific tools to assess self-efficacy levels. Celiac-SE fills this gap.

The Celiac-SE has good psychometric properties, both valid and reliable in detecting specific levels of self-efficacy in celiac disease sufferers in the main spheres of life in which they can experience problems: eating in the workplace, shopping, traveling, eating out, and eating at home with others. The questionnaire is easily and quickly administered (15 min at the most) and determines the degree of specific self-efficacy in diagnosed celiac sufferers following a life-long GFD. Its construction followed scientific procedures in accordance with the recommendations for constructing scales of this type (Bandura, 2006), and was supported by the consensus of patients and experts. A factorial analysis revealed the existence of five factors that coincided with the areas intended to measure specific self-efficacy for following a GFD.

The test shows a high reliability indicating that the items are grouped around the five areas we wish to explore. The tool presents an acceptable concurrent validity, showing positive and significant correlations with the GSES of Baessler & Schwarzer. Although this coefficient is significant, its low value can be explained because the GSES scale measures general self-efficiency, as opposed to the specific self-efficacy measured by our scale.

Food intolerances are becoming increasingly frequent and better diagnosed in today's world. The tool will help patients and professionals alike to improve the formers' adherence to what is, to date, the only treatment available. New intervention models in health place emphasis on patients' responsibility and their empowerment to direct their treatment and improve their quality of life (Holman and Lorig, 2000, 2004; Bodenheimer et al., 2002; Carey and Doherty, 2012). The aim of this research was to provide tools to study these psychosocial factors in order to establish the bases for the design of self-management programs for sufferers of celiac disease (Fueyo-Díaz et al., 2017).

Bandura defines expectation of self-efficacy as “the judgement of one's ability to organize and execute given types of performances” (Bandura, 1997, p. 21), thus clearly distinguishing between expectation of self-efficacy and other psychological constructs such as feelings, outcome expectations, locus of control, etc. Thus, this scale seeks to explore not so much feelings and motivations for following a GFD, but the confidence a person has that he or she will be able to follow it. The contribution of this questionnaire is that it makes available a tool that can measure expectations of self-efficacy in specific situations which are real and frequent and which have their own specific demands, as opposed to measuring general expectation. Furthermore, the scale provides not only a measurement of self-efficacy, but also valuable information about its levels in the various situations in which a celiac disease sufferer moves. From the theoretical viewpoint, therefore, this study lies within the Social Cognitive Theory developed by Bandura (1985) and is a first step toward the future design of self-management programs for the disease of the type developed by Kate Lorig (Bodenheimer et al., 2002) at the University of Stanford. More recently, the research lines of Schwarzer on the HAPA model (Schwarzer et al., 2011) propose a more specific theoretical framework in which to interpret results and develop self-management programs for the disease.

The levels of specific self-efficacy found in this validation study are, in general, high. An explanation for these high scores may be the mastery in GFD management of the sample as participants had more than 8.77 years of experience. It seems self-efficacy beliefs are built during the first year of experience in GFD and reach their highest levels after 5 years on GFD. An explanation for differences for age could be that teens are, somehow, under parental advice at that age and it is between 18 and 35 years old when parents transfer responsibility for the diet and they must face the challenge for managing their diet by themselves when shopping, traveling or at work. These results are consistent with Social Cognitive Theory for which mastery experiences become the most important source of self-efficacy (Bandura, 1997). These relationships need of future research to find out if this scale would predict GFD adherence or quality of life soon after diagnosis.

Some limitations of the study are that it has been developed and validated in Spanish, although we do offer an English version should other researchers wish to validated it in other settings (Appendix). Due to its specific nature and its being closely linked to self-efficacy expectation, it was not considered necessary to study its test-retest reliability, although this could be studied in the future, as could its discriminant validity. Future studies are needed to confirm the factorial structure of the scale as well as to consider an item reduction, e.g., eliminating items 1 or 22 if it is confirmed they load in other factors.

In clinical settings, this scale may be useful to detect patients with low self-efficacy expectations in dealing with GFD and that, therefore, may show worse adherence to diet or worse quality of life. With a cut-off point of 7 for the lowest levels of self-efficacy, we see that around 11.5% present self-efficacy problems in managing their GFDs. These are the patients who could benefit from empowerment programs that can help them to address their illness with lower levels of stress and better life quality.

## Conclusion

Control of the disease through a proper GFD has a direct impact on the celiac patients' perceived quality of life (Casellas et al., 2008, 2015). This tool will, in the future, enable us to explore the expectation of self-efficacy in a specific way, and, hence, its impact on adherence to a GFD and the subsequent quality of life. It should also help in the design of self-management programs for celiac disease and, with the appropriate adaptations, help other sufferers of diseases with dietary restrictions.

## Author contributions

RF-D, SG-S, ÁA-M, RM-B and JS-D were involved in conceptualizing the study, collecting, analyzing the data, and writing the manuscript. GP-N was involved in analyzing the data and the final revision of the work. All authors approved the final submitted version, and all agreed to be accountable for all aspects of the work.

### Conflict of interest statement

The authors declare that the research was conducted in the absence of any commercial or financial relationships that could be construed as a potential conflict of interest.
