# Supplementary material for: Development and Validation of a Specific Self-Efficacy Scale in Adherence to a Gluten-Free Diet
Source: Front Psychol. 2018 Mar 16;9:342. doi: 10.3389/fpsyg.2018.00342 (PMC5864907; doi:10.3389/fpsyg.2018.00342)
Supplement: Supplementary file 1 [file DataSheet1.pdf]

Appendix

Celiac-SE

Intente ponerse en cada una de las situaciones. Algunas preguntas le pueden parecer repetidas pero hacen referencia a distintos ámbitos de su vida.

Si alguna considera que alguna pregunta no le aplica puede dejarla en blanco pero intente contestarlas todas.

Puntúe cada una de las preguntas de 0 a 10 conforme a la siguiente escala, hasta qué punto piensa Vd. que tiene confianza para llevar una dieta sin gluten en las distintas situaciones:

0	1	2	3	4	5	6	7	8	9	10
No me creo capaz en absoluto					Moderadamente capaz					Me creo capaz con toda seguridad

PREGUNTA	CONFIANZA
COMPRA: Algunas personas experimentan problemas para conseguir productos sin gluten a la hora de ir a la compra o para resistir a las tentaciones de comprar y consumir algunos productos que podrían tener gluten. Valore su confianza de 0 a 10 en cada una de las siguientes situaciones:	Confianza<br>0, no me creo capaz en absoluto<br>10, me creo capaz con toda seguridad
1. Cuando tengo que solicitar que limpien las máquinas, utensilios y superficies, si lo creo necesario, por ejemplo, en una charcutería o carnicería, mi confianza para pedirlo es de…	0 ☐ 1 ☐ 2 ☐ 3 ☐ 4 ☐ 5 ☐ 6 ☐ 7 ☐ 8 ☐ 9 ☐ 10 ☐
2. Cuando en la compra tengo que rechazar un producto que no me ofrece seguridad.	0 ☐ 1 ☐ 2 ☐ 3 ☐ 4 ☐ 5 ☐ 6 ☐ 7 ☐ 8 ☐ 9 ☐ 10 ☐
3. Cuando tengo que resistirme a comprar algo en el supermercado muy apetitoso pero que podría contener gluten.	0 ☐ 1 ☐ 2 ☐ 3 ☐ 4 ☐ 5 ☐ 6 ☐ 7 ☐ 8 ☐ 9 ☐ 10 ☐
4. Mi confianza para seguir todas las recomendaciones médicas y de las asociaciones durante la compra es de…	0 ☐ 1 ☐ 2 ☐ 3 ☐ 4 ☐ 5 ☐ 6 ☐ 7 ☐ 8 ☐ 9 ☐ 10 ☐
VIAJES: Para muchos celíacos viajar por su país o por el extranjero supone un reto a la hora de conseguir seguir su dieta sin gluten. Valore, por favor, la confianza de 0 a 10 que tiene para cada una de las siguientes situaciones:	Confianza<br>0, no me creo capaz en absoluto<br>10, me creo capaz con toda seguridad
5. Cuando voy de viaje y tengo que conseguir un menú sin gluten, y no llevo la comida de casa.	0 ☐ 1 ☐ 2 ☐ 3 ☐ 4 ☐ 5 ☐ 6 ☐ 7 ☐ 8 ☐ 9 ☐ 10 ☐
6. Cuanto tengo que conseguir un menú sin gluten al viajar por sitios conocidos, y no llevo la comida de casa.	0 ☐ 1 ☐ 2 ☐ 3 ☐ 4 ☐ 5 ☐ 6 ☐ 7 ☐ 8 ☐ 9 ☐ 10 ☐
7. Cuando tengo que conseguir un menú sin gluten al viajar por sitios desconocidos en España y no llevo la comida de casa.	0 ☐ 1 ☐ 2 ☐ 3 ☐ 4 ☐ 5 ☐ 6 ☐ 7 ☐ 8 ☐ 9 ☐ 10 ☐
8. Cuando tengo que viajar por sitios fuera de mi país pero en los que hablo el idioma y no llevo la comida de casa.	0 ☐ 1 ☐ 2 ☐ 3 ☐ 4 ☐ 5 ☐ 6 ☐ 7 ☐ 8 ☐ 9 ☐ 10 ☐
9. Mi confianza para no saltarme la dieta sin gluten, cuando de turismo en una ciudad, quiero probar los restaurantes o comidas típicas es de…	0 ☐ 1 ☐ 2 ☐ 3 ☐ 4 ☐ 5 ☐ 6 ☐ 7 ☐ 8 ☐ 9 ☐ 10 ☐
10. Seguir mi dieta sin gluten cuando viajo en un tren o en avión y no hay mucho entre lo que elegir.	0 ☐ 1 ☐ 2 ☐ 3 ☐ 4 ☐ 5 ☐ 6 ☐ 7 ☐ 8 ☐ 9 ☐ 10 ☐

COMER CON OTROS EN CASA: En ocasiones, aún cuando come Vd. en casa, se producen situaciones que exigen de confianza para afrontarlas con eficacia a la hora de seguir una estricta dieta sin gluten. Valore tu confianza de 0 a 10 para cada una de las situaciones siguientes:	Confianza<br>0, no me creo capaz en absoluto<br>10, me creo capaz con toda seguridad
11. Vencer la tentación de saltarme la dieta sin gluten cuando tengo la casa llena de cosas apetitosas.	0 ☐ 1 ☐ 2 ☐ 3 ☐ 4 ☐ 5 ☐ 6 ☐ 7 ☐ 8 ☐ 9 ☐ 10 ☐
12. Para rechazar una comida o regalo que puede contener gluten que traen otras personas y me invitan a probarla y no quiero parecer descortés.	0 ☐ 1 ☐ 2 ☐ 3 ☐ 4 ☐ 5 ☐ 6 ☐ 7 ☐ 8 ☐ 9 ☐ 10 ☐
13. Cuando cocino para otros comida que puede tener gluten y deseo acompañarlos.	0 ☐ 1 ☐ 2 ☐ 3 ☐ 4 ☐ 5 ☐ 6 ☐ 7 ☐ 8 ☐ 9 ☐ 10 ☐
14. Cuando alguien me ofrece a probar algo de su plato que puede tener gluten.	0 ☐ 1 ☐ 2 ☐ 3 ☐ 4 ☐ 5 ☐ 6 ☐ 7 ☐ 8 ☐ 9 ☐ 10 ☐
COMER CON OTROS FUERA DE CASA: Comer fuera de casa, con frecuencia, supone un reto para mantener una estricta dieta sin gluten. Valore la confianza que tiene de 0 a 10 en cada una de las situaciones propuestas para seguir una estricta dieta sin gluten:	Confianza<br>0, no me creo capaz en absoluto<br>10, me creo capaz con toda seguridad
15. Para identificarme ante el camarero como celíaco en un restaurante cuando voy solo.	0 ☐ 1 ☐ 2 ☐ 3 ☐ 4 ☐ 5 ☐ 6 ☐ 7 ☐ 8 ☐ 9 ☐ 10 ☐
16. Para identificarme ante el camarero como celíaco en un restaurante cuando voy con amigos.	0 ☐ 1 ☐ 2 ☐ 3 ☐ 4 ☐ 5 ☐ 6 ☐ 7 ☐ 8 ☐ 9 ☐ 10 ☐
17. Para de identificarme como celiaco ante el camarero en un restaurante cuando voy con personas con las que no tengo confianza.	0 ☐ 1 ☐ 2 ☐ 3 ☐ 4 ☐ 5 ☐ 6 ☐ 7 ☐ 8 ☐ 9 ☐ 10 ☐
18. Cuando quiero relajarme y disfrutar de una comida en un tranquilo restaurante.	0 ☐ 1 ☐ 2 ☐ 3 ☐ 4 ☐ 5 ☐ 6 ☐ 7 ☐ 8 ☐ 9 ☐ 10 ☐
19. Para rechazar un plato que pienso que no cumple las condiciones de seguridad suficientes en un restaurante una vez me lo han traído a la mesa.	0 ☐ 1 ☐ 2 ☐ 3 ☐ 4 ☐ 5 ☐ 6 ☐ 7 ☐ 8 ☐ 9 ☐ 10 ☐
20. Para pedir un plato en un restaurante con las condiciones de seguridad suficientes para seguir una dieta sin gluten.	0 ☐ 1 ☐ 2 ☐ 3 ☐ 4 ☐ 5 ☐ 6 ☐ 7 ☐ 8 ☐ 9 ☐ 10 ☐
21. Sacar y comer una comida, que he traído conmigo de casa por si no había menú sin gluten, cuando estoy solo.	0 ☐ 1 ☐ 2 ☐ 3 ☐ 4 ☐ 5 ☐ 6 ☐ 7 ☐ 8 ☐ 9 ☐ 10 ☐
TRABAJO O ESTUDIOS: Algunas personas con celiaquía experimentan dificultades a la hora de manifestar su necesidad de conseguir una comida sin gluten en un entorno laboral o de estudios. Valore de 0 a 10 su confianza para cada una de las siguientes situaciones:	Confianza<br>0, no me creo capaz en absoluto<br>10, me creo capaz con toda seguridad
22. Para identificarme como celíaco en comidas de empresa o estudios	0 ☐ 1 ☐ 2 ☐ 3 ☐ 4 ☐ 5 ☐ 6 ☐ 7 ☐ 8 ☐ 9 ☐ 10 ☐
23. Para conseguir una comida y bebida sin gluten en mi entorno de trabajo o estudios	0 ☐ 1 ☐ 2 ☐ 3 ☐ 4 ☐ 5 ☐ 6 ☐ 7 ☐ 8 ☐ 9 ☐ 10 ☐
24. Para conseguir una comida sin gluten en viajes de empresa o excursiones	0 ☐ 1 ☐ 2 ☐ 3 ☐ 4 ☐ 5 ☐ 6 ☐ 7 ☐ 8 ☐ 9 ☐ 10 ☐
25. Para conseguir una comida y bebida sin gluten en celebraciones de empresa o de estudios.	0 ☐ 1 ☐ 2 ☐ 3 ☐ 4 ☐ 5 ☐ 6 ☐ 7 ☐ 8 ☐ 9 ☐ 10 ☐

Celiac-SE (English translation)

Try to imagine yourself in each of the following situations. Some questions may appear to be repeated, but they refer to different areas of your life.

If you believe that a question is not applicable to you, you can leave it unanswered, but please try to answer all the questions.

Score each of the questions from 0 to 10 according to the following scale, according to how confident you feel about following a gluten-free diet in each of the situations:

0	1	2	3	4	5	6	7	8	9	10
Not able at all					Moderately able					Totally able

QUESTION	CONFIDENCE
SHOPPING: Some people experience problems in finding gluten-free products when shopping or find it hard to resist the temptation to buy and consume some products that may contain gluten. Rate your confidence from 0 to 10 for each of the following situations:	
1. When I have to ask people to clean machines, utensils or surfaces, for example at the butcher's, I feel able to do this.	0 ☐ 1 ☐ 2 ☐ 3 ☐ 4 ☐ 5 ☐ 6 ☐ 7 ☐ 8 ☐ 9 ☐ 10 ☐
2. When shopping I have to reject a product that may not be safe for me.	0 ☐ 1 ☐ 2 ☐ 3 ☐ 4 ☐ 5 ☐ 6 ☐ 7 ☐ 8 ☐ 9 ☐ 10 ☐
3. When I have to resist buying something that looks very appetizing but may contain gluten.	0 ☐ 1 ☐ 2 ☐ 3 ☐ 4 ☐ 5 ☐ 6 ☐ 7 ☐ 8 ☐ 9 ☐ 10 ☐
4. My belief in my ability to adhere to the recommendations of doctors and associations when shopping is …	0 ☐ 1 ☐ 2 ☐ 3 ☐ 4 ☐ 5 ☐ 6 ☐ 7 ☐ 8 ☐ 9 ☐ 10 ☐
TRAVELLING: Many celiac sufferers when travelling around their country or abroad find it a challenge to adhere to their gluten-free diet. Please rate from 0 to 10 how confident you feel in the following situations:	
5. When I'm travelling and I have to find a gluten-free meal and I have not brought my own food.	0 ☐ 1 ☐ 2 ☐ 3 ☐ 4 ☐ 5 ☐ 6 ☐ 7 ☐ 8 ☐ 9 ☐ 10 ☐
6. When I'm travelling in places I know and I have find a gluten-free meal and I have not brought my own food.	0 ☐ 1 ☐ 2 ☐ 3 ☐ 4 ☐ 5 ☐ 6 ☐ 7 ☐ 8 ☐ 9 ☐ 10 ☐
7. When I have to find a gluten-free meal when travelling in unknown places and I have not brought my own food.	0 ☐ 1 ☐ 2 ☐ 3 ☐ 4 ☐ 5 ☐ 6 ☐ 7 ☐ 8 ☐ 9 ☐ 10 ☐
8. When I travel abroad but speak the language and have not brought my own food.	0 ☐ 1 ☐ 2 ☐ 3 ☐ 4 ☐ 5 ☐ 6 ☐ 7 ☐ 8 ☐ 9 ☐ 10 ☐
9. My confidence in not abandoning my gluten-free diet when visiting a city and I want to go to restaurants to try typical dishes…	0 ☐ 1 ☐ 2 ☐ 3 ☐ 4 ☐ 5 ☐ 6 ☐ 7 ☐ 8 ☐ 9 ☐ 10 ☐
10. Following my gluten-free diet on a train or airplane and there is little choice of food.	0 ☐ 1 ☐ 2 ☐ 3 ☐ 4 ☐ 5 ☐ 6 ☐ 7 ☐ 8 ☐ 9 ☐ 10 ☐

EATING AT HOME WITH OTHERS: Even when eating at home there are occasions when I have to be confident that I can effectively follow my strict gluten-free diet. Please rate from 0 to 10 how confident you feel in the following situations:	
11. Overcome the temptation to abandon the gluten-free diet when the house is full of appetizing food and drink.	0 1 2 3 4 5 6 7 8 9 10<br>☐ ☐ ☐ ☐ ☐ ☐ ☐ ☐ ☐ ☐ ☐
12. Rejecting food or a present that may contain gluten because I don't wish to seem rude which other people bring and invite me try.	0 1 2 3 4 5 6 7 8 9 10<br>☐ ☐ ☐ ☐ ☐ ☐ ☐ ☐ ☐ ☐ ☐
13. When cooking a meal for others that may contain gluten and I want to join in.	0 1 2 3 4 5 6 7 8 9 10<br>☐ ☐ ☐ ☐ ☐ ☐ ☐ ☐ ☐ ☐ ☐
14. When someone offers me something to try from their plate and which may contain gluten.	0 1 2 3 4 5 6 7 8 9 10<br>☐ ☐ ☐ ☐ ☐ ☐ ☐ ☐ ☐ ☐ ☐
EATING OUT: Eating out often is a challenge to maintaining a strict gluten-free diet. Rate your confidence, from 0 to 10, in adhering to a strict gluten-free diet in the following situations:	
15. Informing a server in a restaurant that I am a celiac sufferer when on my own.	0 1 2 3 4 5 6 7 8 9 10<br>☐ ☐ ☐ ☐ ☐ ☐ ☐ ☐ ☐ ☐ ☐
16. Informing a server in a restaurant that I am a celiac sufferer when with friends.	0 1 2 3 4 5 6 7 8 9 10<br>☐ ☐ ☐ ☐ ☐ ☐ ☐ ☐ ☐ ☐ ☐
17. Informing a server in a restaurant that I am a celiac sufferer in the company of others who are not in my confidence.	0 1 2 3 4 5 6 7 8 9 10<br>☐ ☐ ☐ ☐ ☐ ☐ ☐ ☐ ☐ ☐ ☐
18. When I want to relax and enjoy a meal in a quiet restaurant.	0 1 2 3 4 5 6 7 8 9 10<br>☐ ☐ ☐ ☐ ☐ ☐ ☐ ☐ ☐ ☐ ☐
19. When refusing a dish that has been brought to my table in a restaurant because I think it may not be sufficiently safe.	0 1 2 3 4 5 6 7 8 9 10<br>☐ ☐ ☐ ☐ ☐ ☐ ☐ ☐ ☐ ☐ ☐
20. When ordering a meal in a restaurant with sufficient guarantees that it is compatible with a gluten-free diet.	0 1 2 3 4 5 6 7 8 9 10<br>☐ ☐ ☐ ☐ ☐ ☐ ☐ ☐ ☐ ☐ ☐
21. When I am alone, taking out and eating food I had prepared at home in case there was no gluten-free option.	0 1 2 3 4 5 6 7 8 9 10<br>☐ ☐ ☐ ☐ ☐ ☐ ☐ ☐ ☐ ☐ ☐
WORK AND STUDIES: Some celiac sufferers experience difficulties when they have to express their need to eat a gluten-free meal in the workplace or where they study. Rate from 0 to 10 how confident you feel in the following situations:	
22. When identifying myself as a celiac sufferer in the business or students' meal.	0 1 2 3 4 5 6 7 8 9 10<br>☐ ☐ ☐ ☐ ☐ ☐ ☐ ☐ ☐ ☐ ☐
23. Finding gluten-free food and drink in the work or study place.	0 1 2 3 4 5 6 7 8 9 10<br>☐ ☐ ☐ ☐ ☐ ☐ ☐ ☐ ☐ ☐ ☐
24. Finding a gluten-free meal on a business trip or on an excursion.	0 1 2 3 4 5 6 7 8 9 10<br>☐ ☐ ☐ ☐ ☐ ☐ ☐ ☐ ☐ ☐ ☐
25. Finding gluten-free food and drink at business or student celebrations.	0 1 2 3 4 5 6 7 8 9 10<br>☐ ☐ ☐ ☐ ☐ ☐ ☐ ☐ ☐ ☐ ☐
